# New concepts in the roles of AMPK in adipocyte stem cell biology

**DOI:** 10.1042/EBC20240008

**Published:** 2024-11-18

**Authors:** Alice E. Pollard

**Affiliations:** Institute of Clinical Sciences, Faculty of Medicine, Imperial College London, London, U.K.

**Keywords:** Adipose, AMPK, leptins, lipid metabolism

## Abstract

Obesity is a major risk factor for many life-threatening diseases. Adipose tissue dysfunction is emerging as a driving factor in the transition from excess adiposity to comorbidities such as metabolic-associated fatty liver disease, cardiovascular disease, Type 2 diabetes and cancer. However, the transition from healthy adipose expansion to the development of these conditions is poorly understood. Adipose stem cells, residing in the vasculature and stromal regions of subcutaneous and visceral depots, are responsible for the expansion and maintenance of organ function, and are now recognised as key mediators of pathological transformation. Impaired tissue expansion drives inflammation, dysregulation of endocrine function and the deposition of lipids in the liver, muscle and around vital organs, where it is toxic. Contrary to previous hypotheses, it is the promotion of healthy adipose tissue expansion and function, not inhibition of adipogenesis, that presents the most attractive therapeutic strategy in the treatment of metabolic disease. AMP-activated protein kinase, a master regulator of energy homeostasis, has been regarded as one such target, due to its central role in adipose tissue lipid metabolism, and its apparent inhibition of adipogenesis. However, recent studies utilising AMP-activated protein kinase (AMPK)-specific compounds highlight a more subtle, time-dependent role for AMPK in the process of adipogenesis, and in a previously unexplored repression of leptin, independent of adipocyte maturity. In this article, I discuss historic evidence for AMPK-mediated adipogenesis inhibition and the multi-faceted roles for AMPK in adipose tissue.

## Obesity and metabolic diseases: the role of the adipocyte

Recent data from the World Obesity Atlas 2023 highlights the continuing increase in obesity worldwide, with a predicted overweight and obesity rate of over 50% by 2035 [1], despite significant advances in both understanding and in weight-loss therapy [2,3]. Of particular concern is the rise in cases of obesity affecting children 5–19 years of age, with an estimated 20% of boys and 18% of girls predicted to be overweight or obese by 2035 [1]. Amongst the perceived barriers there remains a lack of political interest and action, lack of appropriate health care professional training and, most importantly for this discussion, obesity not being recognised as a disease [1].

Obesity is a complex, multifactorial and non-linear condition, characterised primarily by excess adipose (fat) tissue leading to abnormal weight gain, measured by body mass index (BMI), fat percentage and the waist-to-hip ratio [4,5]. Obesity is a potent risk factor for many well-known life-threatening diseases, known as comorbidities, including Type 2 diabetes (T2D) [6,7], cardiovascular disease (CVD) [8], cancer [9], stroke [10,11] and metabolic-associated fatty liver disease (MAFLD). In the last decade, studies have begun to evaluate the effects of obesity on other, lesser associated diseases, including androgen excess (e.g., polycystic ovary syndrome; PCOS) [12–14], Type 1 diabetes (T1D) [15,16], respiratory infections (SARS-CoV2) [17,18] and infertility [19–22]. Combined, these fields highlight the significant impact of obesity on organismal health, disease resistance, mental wellbeing and our ability as a species to thrive in future generations.

However, the incidence of comorbidities within the obese population is highly variable, with noted differences attributed to biological sex, age, culture, geographical location, income status and education [1,22–27]. Even within a demographic, the development of comorbidities is heterogeneous, with some individuals presenting with severe metabolic disturbance, whilst others remain comparatively healthy [28–30]. A deeper understanding of the cause-effect relationship between excess adiposity, adipose tissue dysfunction and the incidence of metabolic disease is therefore required.

### Adipogenesis: to be or not to be (an adipocyte)

**Figure 1:**
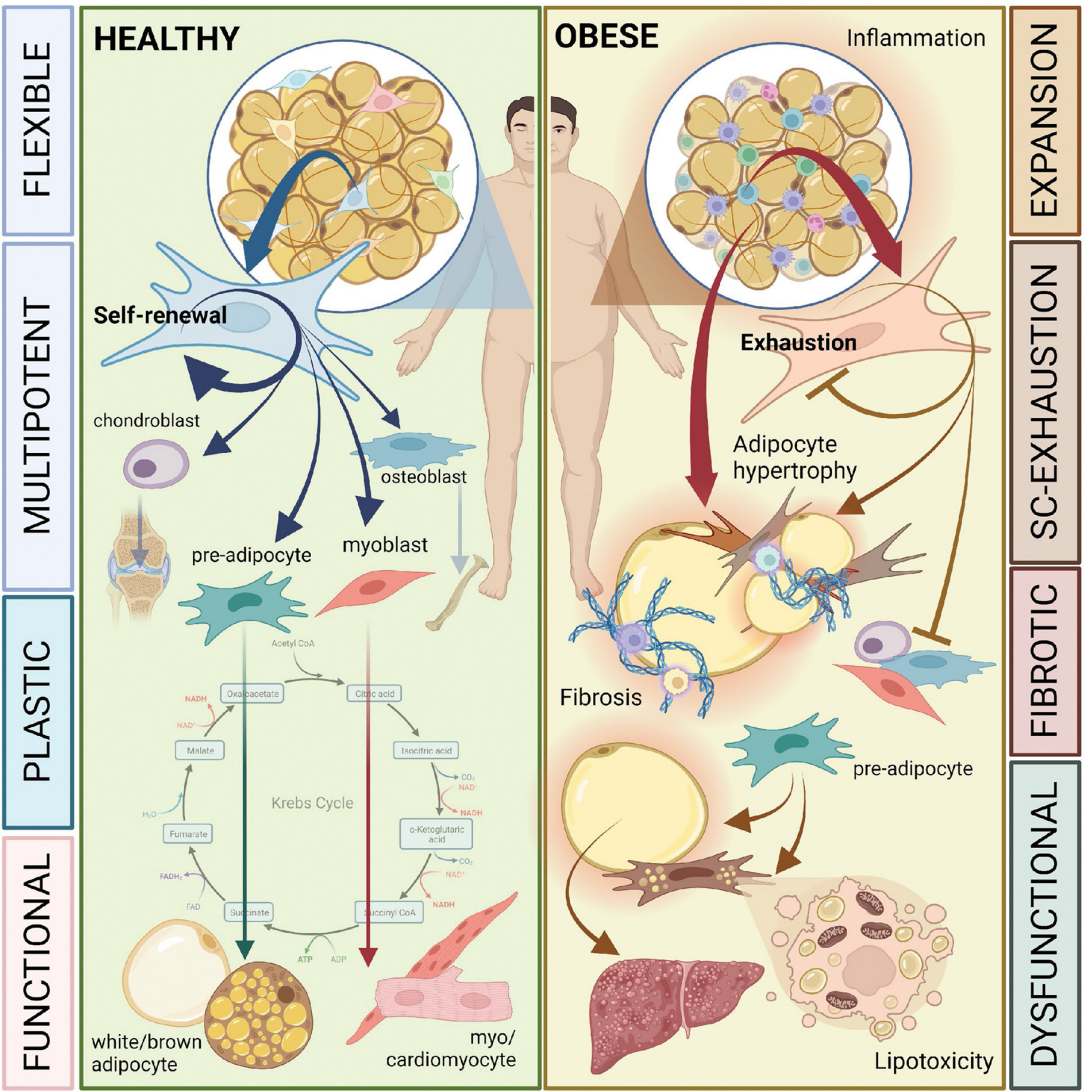
Adipose stem cells in health and metabolic disease Healthy adipose tissue contains a multitude of cell types, creating a hub of cross-talk required for tissue maintenance, expansion and whole organism energy homeostasis. These cell populations include adipocytes, immune cells, vascular endothelium, innervating neuronal cells and mesenchymal stem cells. The development of new adipocytes from the latter, known as adipogenesis, is vital for the storage of energy in the form of triglycerides. In addition to their capacity for adipogenesis, adipose (derived) stem cells (ASCs/ADSCs) are considered multipotent, with the ability to self-renew, and to divert into other cell lineages, including osteo-, chondro- and myogenic trajectories. Loss of ADSC stemness is an emerging hallmark of metabolic disease. In the context of obesity, adipose tissue is forced to expand to provide storage of excess nutrients, a process that exerts mechanical, endocrine and metabolic stress on adipose tissue depots. Expansion through adipocyte hypertrophy and hyperplasia with insufficient vascularisation leads to hypoxia, pro-inflammatory cytokine release and adipocyte death, resulting in the infiltration of, and activation of resident immune cells. The over-recruitment of ADSCs, in conjunction with exposure to the resulting tissue microenvironment, leads to ADSC exhaustion, loss of self-renewal and, in some cases, the promotion of myofibroblast differentiation, in which extracellular matrix-producing cells proliferate and deposit ECM, causing adipose tissue fibrosis. The concurrent loss of storage capacity in the adipose depot results in lipid storage in peripheral tissues, where it proves cytotoxic. Accumulation of lipid in the liver results in non-alcoholic fatty liver disease (NAFLD), a precursor to non-alcoholic steatohepatitis (NASH) and hepatocellular carcinoma (HCC). Increased circulating lipids transported by low-density lipoproteins (LDL) are readily taken up by macrophages, leading to foam cell formation, fatty streak development and, ultimately, atherosclerosis. Figure created with BioRender.com.

A turning-point in our understanding of obesity came with the observation that it is impaired adipose tissue function, not merely adipose tissue mass, that is a driving factor in pathogenesis [31–33]. Adipose tissue plays a central role in the safe storage of excess energy in the form of triglycerides, in the secretion of adipokines such as leptin, adiponectin and in the response to insulin [31,34]. As such, adipose tissue is a key mediator of whole organism energy balance. Subcutaneous adipose tissue expansion, by both adipocyte hypertrophy and hyperplasia from resident adipose stem cells (ASCs), is essential for response to nutritional excess, provides protection against lipotoxicity in vital organs (e.g., liver) and prevents accumulation of lipid in visceral depots, such as in pericardial and omental adipose tissue [35–39] (Figure 1). The importance of healthy adipose tissue expansion is highlighted not only in obesity but also in a group of disorders known as lipodystrophies, a in which adipose tissue is either partially or fully absent [40,41]. Here, metabolic abnormalities such as early onset insulin resistance, leptin deficiency, T2D, hepatic steatosis and cardiovascular disease are prevalent. Both obesity- and lipodystrophy-associated adipose tissue dysfunction has been attributed, in part, to a loss or dysregulation of adipose stem cell function [41].

Though the existence of **a**dipocyte **p**recursors/**a**dipose-**d**erived **s**tem **c**ells (**AP/ASPC/ASC/ADSCs**), has been acknowledged for some time [42], the introduction of cell surface marker-based flow cytometry/fluorescence-activated cell sorting (FACS), high-resolution imaging and, more recently, single-cell RNA sequencing has facilitated the identification of many sub-populations with lineage- and context- dependent functionality [43–52]. Several studies have confirmed, using lineage-dependent reporter mouse models and trajectory analysis, that heterogeneity amongst ASCs is hierarchical, demonstrated in the case of Dpp4+/Pi16+ cells giving rise to Icam1+ and Cd142+ populations, each with unique gene signatures, defined by Merrick et al., in 2019 [35]. The influence of sex, age, environmental stimuli and disease status on these populations is yet to be fully explored. ASCs in human adipose tissue are also heterogeneous, with key depot-specific lineages, trajectories and distributions in diseased state. The publication of the adipose stem cell atlas by Emont et al., in 2022 demonstrated the key overlapping pools of interstitial fibro-adipogenic precursors, committed pre-adipocytes and regulatory adipocyte lineages, with the addition of a population associated with T2D (hAd7) [53]. This new tool adds to an extensive collection of literature exploring the diversity of ASC and adipocyte populations.

The development of new adipocytes from resident precursor cells is known as adipogenesis. Adipogenesis is initiated by exogenous stimuli, including insulin, growth factors, glucocorticoids and other hormones, as well as bone morphogenic proteins (BMPs) [54–56], early b-cell factors (EBF1/2) [57–59] and both endogenous (fatty acid metabolites, prostaglandins) and synthetic modulators of master adipogenic transcription factor PPARγ [60]. Expression of PPARγ, together with adipogenic transcriptional regulator CCAAT/enhancer-binding protein (C/EBP) α is induced by C/EBPβ and δ, and plays a key role in the initial phase of adipocyte commitment, known as mitotic clonal expansion (MCE) [61]. The action of PPARγ serves to induce genes involved in insulin sensitivity, lipid accumulation, such as the sterol response element binding proteins (SREBP) -1a, -1c -2, and those responsible for the generation and secretion of adipokines [62,63]. Absence of, or expression of, dominant-negative PPARγ results in complete or partial lipodystrophy respectively, whilst activation of PPARγ leads to insulin sensitization and adipose tissue expansion [60]. Targeting PPARγ with the thiazolidinedione (TZD) rosiglitazone, however, is associated with increased risk of CVD, congestive heart failure and myocardial infarction, as well as increased general mortality, with side effects including weight gain, edema and osteoporosis [60,64]. The latter has been attributed to the inhibitory effect of PPARγ on osteoblast differentiation, with simultaneous promotion of osteoclastogenesis (bone resorption) resulting in bone loss in rodent models [65,66]. This observation highlights the need for tissue/cell-specific evaluation of compounds targeting key regulators of stem cell fate, with appropriate models to test effects on lineage determination and potential long-term effects on tissue-resident stem cell populations, such as ASCs. Other glitazones, with reduced CVD risk, still face safety warnings and drug withdrawals, suggesting that targeting of PPARγ in this way may not be therapeutically viable, and further research is required to find new ways to promote insulin sensitization in adipose tissue [60]. Several PPARγ-responsive factors, such as fibroblast growth factor (FGF)1 and 21 have been reported to exert beneficial effects in adipose tissue, with improved circulating lipids and glycemic control, whilst avoiding the aforementioned side-effects of TZDs [67–69]. As with TZDs, known to increase the AMP:ATP ratio, the actions of FGF21 were thought to be, in part, due to activation of the evolutionarily conserved metabolic regulator: AMP-activated protein kinase (AMPK) [70]. However, studies using AMPK β1/2 knock-out mice have confirmed that the glucose- and lipid-lowering effects of FGF21, both in adipose tissue and in liver, are independent of AMPK [71]. Indeed, many effects previously attributed to AMPK activation, using non-specific or indirect activation strategies (e.g., Metformin, 5-aminoimidazole-4-carboxamide ribonucleoside [AICAR]), are now known to be independent of AMPK [72–75]. This paradigm shift is largely due to the development of more specific small molecule AMPK activators, such as 991 [76–81] and, more recently, BI-9774 [82,83], and the increased use of AMPK-KO systems, both *in vitro* and *in vivo*. It is, and will continue to be, important for the scientific community to re-evaluate mechanisms attributed solely to an activation of AMPK, as have now been identified in adipose tissue and liver, using these compounds. Additionally, increased use of these small molecules at physiologically relevant concentrations may reveal pathways regulated by AMPK previously unidentified due to AMPK-independent pathway activation.

## AMPK and adipocyte metabolism: a historical perspective

**Figure 2:**
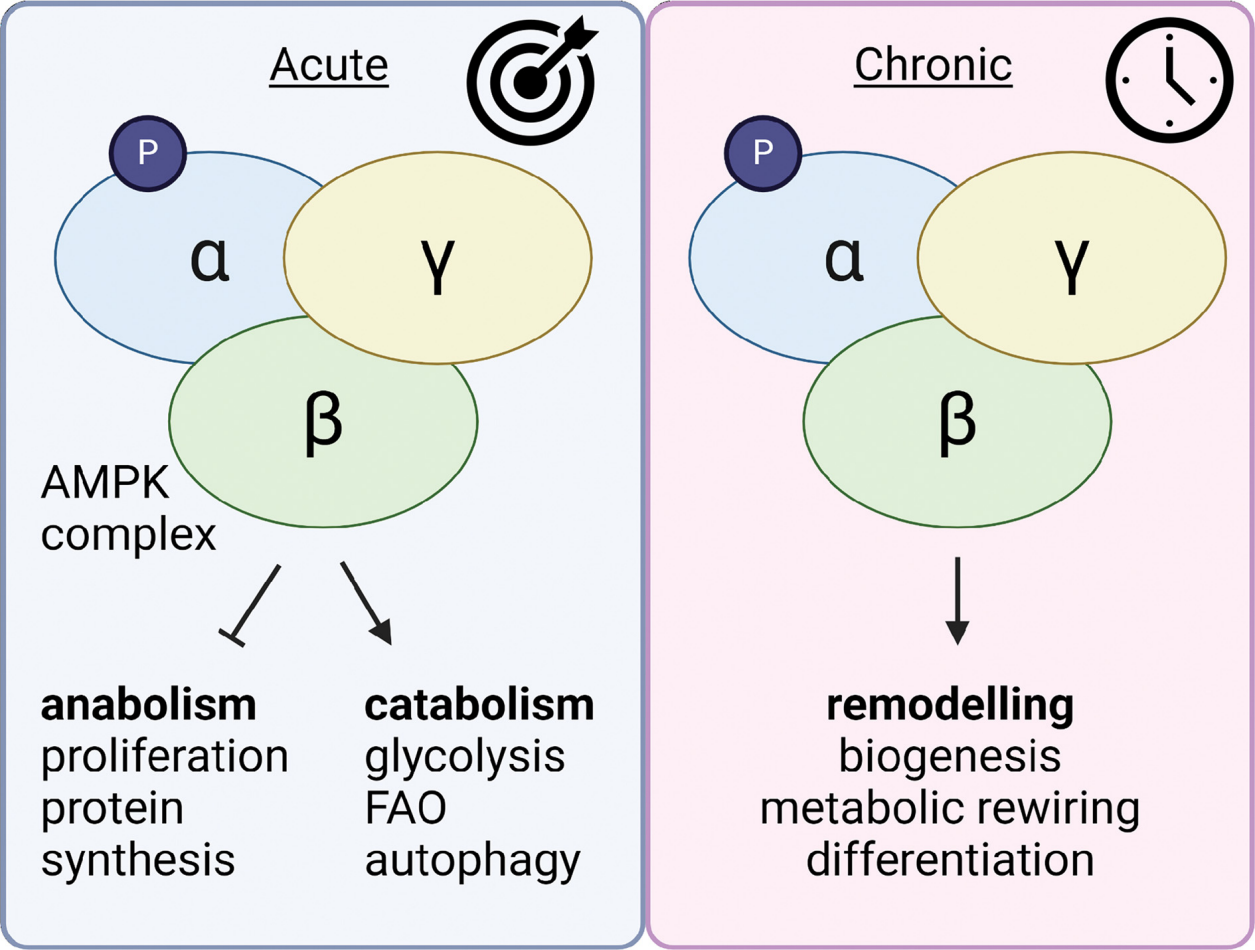
AMP-activated protein kinase: friend or foe? AMPK is an evolutionarily conserved, heterotrimeric serine/threonine kinase, comprised of a catalytic alpha subunit (a1, a2) and regulatory beta (b1, b2) and gamma (y1, y2, y3) subunits. AMPK is phosphorylated by upstream kinases CAMKK2 and LKB1 at threonine 172 of the alpha subunit. This phosphorylation site is subject to continuous dephosphorylation by phosphatases, thus requiring a conformational change to physically prevent deactivation. This alteration is introduced primarily through the binding of AMP or ADP, in place of ATP, in the nucleotide binding sites on the gamma subunit. Therefore, activation of AMPK is driven primarily by an increase in the AMP/ADP:ATP ratio, a reflection of cellular energy status. During periods of energetic stress, such as starvation, glucose deprivation or mitochondrial toxicity, cellular ATP levels fall, and AMP/ADP levels rise. AMPK activation under these conditions results acutely in the inhibition of ATP-consuming pathways, such as lipid and protein synthesis, through phosphorylation of acetyl Co-A carboxylase (ACC) and mTORC1, respectively. To replenish ATP, AMPK promotes ATP-generating pathways; glycolysis, fatty acid oxidation and autophagy, thus restoring cellular energy homeostasis. The effects of chronic AMPK activation are less well established, largely due to lack of specific, bioavailable small-molecule activators, tissue-specific downstream targets, cellular epigenetic status and cell/organ cross-talk. It is widely accepted that chronic AMPK activation, most notably through exercise, leads to the biogenesis of energy-producing organelles, namely the mitochondria and the lysosome, which will, in the event of future ATP depletion, provide enhanced ATP-generating capacity. It is for this reason, among many others, that AMPK has provided the field with an enticing pharmaceutical target for metabolic diseases, through the promotion of mitochondrial respiratory capacity, increased insulin sensitivity and inhibition of lipid accumulation. Figure created with BioRender.com

AMPK is widely regarded as a master regulator of energy homeostasis and is activated in response to energetic stress. Acute AMPK activation leads to cessation of ATP-consuming pathways, such as lipogenesis, and initiates catabolic pathways that serve to replenish cellular ATP levels, including autophagy, mitophagy and glucose uptake [84]. In addition to these short-term effects, AMPK activation leads to biogenesis of organelles required for ATP generation, the mitochondrion and the lysosome [81,83,85,86]. This occurs through phosphorylation of folliculin-interacting protein (FNIP)1, leading to FNIP1-mediated PGC1α activation and the induction of mitochondrial and lysosomal gene expression [81]. Many downstream targets of AMPK have been identified, some ubiquitous and others cell/tissue-specific (Figure 2).

In adipose tissue, AMPK has been implicated in many cellular processes, from lipid accumulation to transcriptional regulation of adipogenesis. Non-adipose tissue activation of AMPK is strongly associated with prevention of lipid accumulation due to its role in the regulation of acetyl coenzyme A (CoA) carboxylase (ACC) [87]. The phosphorylation of ACC by AMPK prevents the conversion of acetyl CoA to malonyl CoA, inhibiting lipogenesis. Similarly, AMPK activation restrains cholesterol synthesis through 3‐hydroxy‐3‐methylglutaryl (HMG) CoA reductase (HMGCR) phosphorylation [88,89], and through the phosphorylation of sterol-response element binding protein (SREBP)1-c at Ser372, preventing its cleavage and subsequent translocation to the nucleus [90–92]. However, many roles reported for AMPK in adipocytes are controversial.

Increasing AMPK activity in mature adipocytes has been reported to both increase and inhibit lipolysis, though recent studies favor the latter, through the phosphorylation and inhibition of hormone sensitive lipase (HSL) translocation to lipid droplets [93]. Through phosphorylation of the RAB GTPase-activating protein (GAP) TBC1 domain family member 1 (TBC1D1) and 4, AMPK facilitates, and is essential for, glucose uptake through glucose transporter (GLUT) 4 translocation to the plasma membrane in skeletal muscle [94–97]. However, in adipocytes, AMPK has been shown to be dispensable for this process. Indeed, stimulation of 3T3-L1 derived adipocytes with AICAR leads to an inhibition of insulin-stimulated glucose uptake, suggesting an opposing role for AMPK in this pathway in adipocytes [98]. A later study, in contrast, demonstrated the promotion of GLUT4 translocation by AICAR and dinitrophenol (DNP) (non-specific). A caveat with historical studies such as these, looking into the role of AMPK in adipocytes, is the use of non-specific AMPK activators, such as AICAR, which may produce effects entirely independent of AMPK. A comprehensive review of the role of AMPK in canonical adipose metabolism, published in 2023, provides a more detailed perspective of these studies [99]. Many *in vivo* studies have utilized mature white and brown adipose-specific (Adiponectin-Cre) and whole-body AMPK knock-out mice to examine the role of AMPK in mature adipose. In both α1/α2 [100] and β1/β2 KO models [101], detrimental effects on mitochondrial health are observed, attributed to inhibition of both mitochondrial turnover through ULK1 and through poor mitochondrial biogenesis (PGC1α). These mice exhibit cold-intolerance, through insufficient non-shivering thermogenesis in brown or white adipose tissue [101]. These, in addition to many others examining isoform-specific KO effects, though informative, give little information on the role of AMPK in adipocyte stem cells, or in adipose tissue development, and further studies utilising ADSC-specific models are required.

## The controversial role of AMPK in adipogenesis

Given that, for many decades, the expansion of adipose tissue was considered pathological in itself, it is unsurprising that many therapeutic strategies for prevention of obesity have focused on the inhibition of adipogenesis. Initial studies identifying AMPK as a potential regulator of adipogenesis emerged in 2001, when Habinowski and Witters described a profound inhibition of 3T3-L1 adipocyte differentiation by AICAR [102]. Here, AMPK activation during adipocyte commitment coincided with a blockade of adipogenic transcription factor (C/EBPα and PPARγ) expression, a repression of MCE, C/EBPβ retention and suppression of late adipogenic markers such as ACC and fatty acid synthase (FAS). Importantly, this effect was only observed if AICAR was added during adipogenic induction. C/EBPβ is required for the transactivation of C/EBPα and subsequent PPARγ expression, both of which are reduced by AICAR when added from adipogenic day 1 [102]. In adipose precursors, AICAR did not increase C/EBPβ expression, nor did it inhibit the relative increase in response to adipogenic induction. Together these results suggested AICAR may act to prevent C/EBPβ activity rather than expression, owing to its retention through to later stages under these conditions.

Later work identified a role for AMPK in the prevention of MCE, with subsequent inhibition of adipogenesis [103]. This work employed the small molecule AMPK activator A-769662 at millimolar concentrations, where inhibition was complete, though significant AMPK activation was achieved at 300 μM, where no significant effect on differentiation was observed. Given that, at high concentrations, A-769662 is now known to exert off-target effects, it is most likely that this inhibition of differentiation is not attributable entirely to AMPK [104]. Indeed, at 300 μM, the effect on transcription factor activity is modest, whilst at the concentration used to draw mechanistic conclusions, the repression is considerable. Additional studies using resveratrol-mimetics RSVA314 and RSVA405 as indirect AMPK activators report an inhibition of adipogenesis, in 3T3-L1 cells through interference with MCE [105]. As with A-769662, the authors show concentration- and time-dependent effects of these compounds, though with the latter study, the repression of adipogenesis at the highest concentration, where AMPK activation is maximal, achieves approximately 50% reduction in oil red O staining [105]. As seen by Habinowski and Witters, this repression is only observed if AMPK is active during commitment, with no effect if added at a later stage [102]. The anti-adipogenic effects of many other compounds are documented, with effects attributed most commonly to activation of AMPK and prevention of MCE, inhibition of transcription factor expression and repression of lipid accumulation. In each case, the lowest dose of compound required to achieve maximal AMPK activation as measured by ACC phosphorylation achieves only modest reductions in adipogenesis, whilst higher concentrations are often taken forward into mechanistic studies.

Our recent study [82] evaluated activation of AMPK in all stages of adipogenesis, building on previous studies using indirect activators, as well as those using small molecules at high concentrations associated with AMPK-independent signalling. Using a novel small molecule AMPK activator BI-9774 [83,106], we demonstrated a time- and concentration-dependent effect of AMPK activation, confirming the opposing roles of AMPK in stem cell lineage commitment and in adipocyte maturation [82]. Several studies have indicated that AMPK activation may inhibit adipogenesis through modulation of the transforming growth factor (TGF) β-signalling pathway, and through Wingless and Int-1 (Wnt) mediated β-catenin nuclear activity. AICAR has been shown to inhibit adipogenesis, in part, through Wnt/ β-catenin mediated repression of adipogenic gene expression. However, despite observing the well-known repression of TGFβ protein expression, our study concluded that specific AMPK activation with BI-9774 does not increase nuclear β-catenin, nor does it affect SMAD phosphorylation. These studies taken together suggest the effects of AICAR on Wnt/ β-catenin signalling is AMPK-independent.

### AMPK and leptin in adipocyte metabolism

AMPK has often presented an attractive target in adipocytes due to the respective effects of circulating factors to stimulate AMPK activity, then associated with beneficial metabolic adaptations, including mitochondrial biogenesis, improved lipid handling and the suppression of pro-inflammatory cytokines associated with obesity. The adipokine adiponectin (Adipoq) has been reported to activate AMPK in the liver, through interaction with the adiponectin receptor AdipoR1, leading to a reduction in gluconeogenesis through inhibition of phosphoenolpyruvate carboxykinase (PEPCK) and glucose 6-phosphatase (G6Pase) transcription [107]. In human adipocytes, the effects of adiponectin on several secreted factors, including the interleukin IL-6, were mimicked by the administration of AICAR, along with an increase in the secretion of adiponectin itself. Conclusions from these studies suggest a role for AMPK in the suppression of pro-inflammatory cytokine release and preservation of healthy adipocyte metabolism [108]. Additional studies conducted in 3T3-L1 adipocytes highlight a role for Sirtuin (SIRT) 1, an NAD^+^-dependent deacetylase and a known target of AMPK [109] in the inhibition of adipogenesis through its regulation of PPARγ and C/EBPβ, whilst promoting mitochondrial biogenesis through PPARγ-coactivator α (PGC1α), also a well-established target of AMPK [81]. Activation of this pathway was suggested as a regulatory mechanism for several secreted factors, including the hormone leptin, a key mediator of systemic energy homeostasis through regulation of appetite [110]. In our recent study, using BI-9774 we establish a role for AMPK in the suppression, rather than promotion, of leptin secretion [82], independent of the effects of AMPK activity on adipogenesis. The regulation of leptin by AMPK in adipocytes is uncharacterised, despite numerous studies identifying AMPK as a mediator of leptin signalling in the central nervous system and in peripheral tissues [111]. Leptin, a peptide hormone encoded by the *ob* gene, is produced by mature adipocytes, and regulates satiety, behaviour and energy homeostasis via its receptor *LepRb* [111]. Leptin receptors are expressed by many cell types, though its principal site of action is in the brain, where it acts as an indicator of peripheral energy storage, with a decline in circulating leptin seen as a starvation signal. Excess adiposity, leading to a proportional increase in leptin production by mature adipocytes, results in high circulating leptin levels. In turn, elevated serum leptin is thought to drive, in part, leptin resistance in the central nervous system (CNS) [111].

Leptin has been shown to activate AMPK in skeletal muscle, leading to increased fatty acid oxidation, whilst inhibiting lipogenesis through phosphorylation of ACC [112]. In contrast, leptin inhibits AMPK activity in rat hypothalamus [113,114], leading to an increase in food intake. The leptin gene (*ob*) promotor region contains binding sites for C/EBPα, though β and δ isoform co-transfection can stimulate leptin transcription [115]. Additional binding sites for specificity protein (Sp)1 and lipid transfer protein (LP)1 and SREBP1-c have been identified, presenting a number of potential regulatory pathways through which leptin may be regulated in adipocytes [115]. Historically, insulin has been shown to stimulate leptin expression and secretion in subcutaneous, but not omental adipocytes through phosphatidylinositol 3-kinase (PI3K), Akt and mammalian target of rapamycin (mTOR) [116–118]. As AMPK is known to inhibit mTOR complex 1 (mTORC1) through phosphorylation of Raptor and via activation of Tuberous sclerosis (TSC) 2 [119–121], it is possible that this may be a mechanism by which AMPK inhibits leptin, when in the presence of insulin as in the culture of adipocytes *in vitro*. Additional mechanisms may lie in the known regulation of SREBP1c cleavage by AMPK, which would also result in a reduction in leptin expression. Further studies focusing on the role of AMPK in the regulation of leptin may provide alternative therapies in instances of hyperleptinemia, and in the prevention of leptin resistance when adipose tissue expands.

### Future perspectives: a new era in adipocyte metabolism?

**Figure 3:**
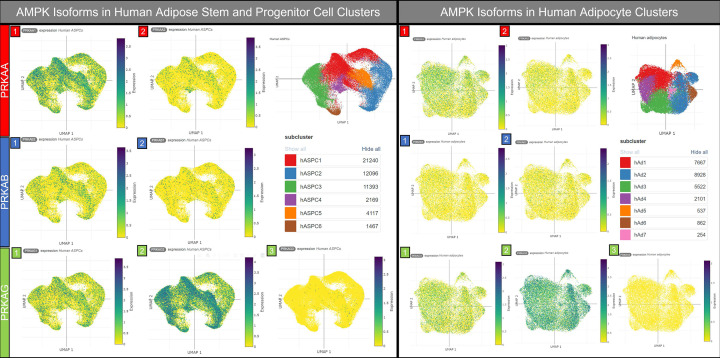
Distribution of AMPK isoforms in human ADSCs and adipocytes The introduction of single-cell and single nuclei sequencing has revealed extensive heterogeneity in human adipose stem cell and adipocyte populations. Several studies have taken large numbers of biological replicates, human and murine, comparing the transcriptome of these subpopulations, creating vast publicly available data sets. Perhaps the most complete of these is the single cell atlas of human and mouse white adipose tissue (Emont et al., 2022, Nature) in which single-cell and whole-nucleus sequencing of subcutaneous and visceral adipose tissue, encompassing mature adipocytes, ADSCs (ASPCs), vascular and immune cells, from lean and obese subjects. Though AMPK activity is not often regulated at the transcriptional level, these data can be used to identify predominant AMPK isoform expression in adipocytes and ADSC/ASPCs, which may inform drug discovery. As highlighted in this collated data from the Single Cell Portal, AMPKγ2 is highly expressed in both ASPCs (left) and white adipocytes (right), separated into subclusters hASPC1-6 and hAd1-7, respectively.

Though the role of AMPK in adipogenesis is controversial, with perspectives altered by new generations of AMPK activators, AMPK still presents an attractive therapeutic target for the promotion of insulin sensitivity, glucose uptake and metabolic regulation in adipocytes. Previous studies identified considerable differences between rodent and human AMPK isoform distribution and relative activity in adipose tissue, and in isolated adipocytes [99,122]. New technologies, including single-cell RNA and single-nuclei sequencing, spatial transcriptomics and single-cell proteomics have opened doors to identify subpopulations of cells in which AMPK may differentially regulate downstream signalling. Though AMPK activity is rarely regulated at the transcriptional level, the differential isoform expressions observed in ADSCs (ASPCs) and human adipocytes may hint at complex-specific signalling pathways, particularly given the high expression of AMPKγ2 (Figure 3, data obtained from Single Cell Portal [53]), which is known to be more sensitive to small molecule activation [78]. This may prove vital in the development of compounds targeting or avoiding specific AMPK complexes, to avoid detrimental effects on other organs known to be susceptible, such as the heart [78,123,124] and kidney [125]. The diversity of adipocyte lipid and glucose metabolism seen by Backdahl et al. [126], in human subcutaneous WAT by spatial transcriptomics may well prove to coincide with AMPK signalling, and this will undoubtedly be explored in the future. Given that the promotion of healthy adipose expansion has overtaken the desire for a repression of adipogenesis in the treatment of obesity and associated metabolic disorders, it may be that AMPK presents a more attractive target in this context, than in the inhibition of adipocyte differentiation, now known to drive, rather than alleviate, adipose tissue dysfunction.

## Summary

- Inhibition of adipogenesis is a driver of adipose tissue dysfunction, leading to fibrosis, inflammation and development of the metabolic syndrome.
- Physiological AMPK activation reduces, but does not inhibit adipogenesis, with the majority of effects constrained to adipogenic initiation.
- AMPK activation suppresses leptin expression, independent of adipogenesis or adipocyte maturity.
- Therapeutic strategies promoting healthy stem cell differentiation and improvement in terminal cell function are a priority.
- AMPK activators may be beneficial in the promotion of healthy adipocyte differentiation and metabolism.

## Open Access

Open access for this article was enabled by the participation of Imperial College London in an all-inclusive *Read & Publish* agreement with Portland Press and the Biochemical Society under a transformative agreement with JISC.

## Abbreviations

ACC: acetyl coenzyme A carboxylase
AMPK: AMP-activated protein kinase
CNS: central nervous system
FAS: fatty acid synthase
GAP: GTPase-activating protein
HMG: 3‐hydroxy‐3‐methylglutaryl
HSL: hormone sensitive lipase
LP: lipid transfer protein
mTOR: mammalian target of rapamycin
PEPCK: phosphoenolpyruvate carboxykinase
PI3K: phosphatidylinositol 3-kinase
SIRT: Sirtuin
SREBP: sterol-response element binding protein
TBC1D1: TBC1 domain family member 1
TGF: transforming growth factor
TSC: Tuberous sclerosis
